# 2329 Associations between inflammatory markers and negative symptoms in individuals with schizophrenia: Converging evidence

**DOI:** 10.1017/cts.2018.46

**Published:** 2018-11-21

**Authors:** David Goldsmith, Robert Cotes, Brian J. Miller, Michael T. Treadway, Elaine F. Walker, Andrew H. Miller

**Affiliations:** 1 Department of Psychiatry and Behavioral Sciences, Emory University; 2 Department of Psychiatry and Health Behavior, Augusta University; 3 Department of Psychology, Emory University

## Abstract

OBJECTIVES/SPECIFIC AIMS: Negative symptoms of schizophrenia, including motivational deficits, social withdrawal, poverty of speech, decreased emotional reactivity, and psychomotor retardation, have been shown to be most predictive of functional impairment and poor outcome in patients with schizophrenia. Furthermore, these symptoms tend not to be responsive to antipsychotic medications. Inflammation could be one mechanism underlying these difficult to treat symptoms. METHODS/STUDY POPULATION: Three cohorts of patients, reflecting different phases of disease, were studied. One cohort was comprised of a sample of patients with deficit schizophrenia (characterized by primary and enduring negative symptoms; n=17), nondeficit patients (n=39), and healthy controls (n=28). ANOVA and multivariate general linear models were used to compare groups, and linear regression models were used to examine relationships between inflammatory cytokines and negative symptoms. The second cohort was comprised of 80 individuals at clinical high risk for psychosis from the North American Prodromal Longitudinal Study. Linear regression models examined the relationship between baseline inflammatory markers and subsequent negative symptoms at follow-up visits up to 2 years. The third cohort consisted of patients with treatment-resistant schizophrenia (TRS) on clozapine (n=10). Correlations were performed to examine relationships between inflammatory markers and negative symptoms. In a subgroup of patients from this third sample, resting state functional connectivity analyses were performed on fMRI data to explore relationships between inflammatory markers and connectivity in brain reward circuitry. RESULTS/ANTICIPATED RESULTS: In a sample of patients with the deficit syndrome of schizophrenia (n=17), a subtype of the disorder characterized by primary and enduring negative Symptoms, tumor necrosis factor (TNF) was significantly increased relative to nondeficit patients (n=39) and healthy controls (n=28; *F*_2,57_=3.51, *p*=0.036), and predicted total negative symptoms (β=0.31, *p*=0.012), alogia (β=0.30, *p*=0.024), and blunted affect (β=0.31, *p*=0.018) items of the Positive and Negative Symptom Scale in linear regression models while controlling for antipsychotics. In another sample of individuals at clinical-high risk for psychosis (n=80), baseline concentrations of TNF significantly predicted negative symptoms, including anhedonia, apathy, and loss of interest in linear regression models, at the 6-month (β=0.25, *p*=0.011) and 12-month follow-up (β=0.39, *p*=0.001). Interleukin (IL)-1 receptor antagonist also predicted these symptoms at the 6-month follow-up (β=0.21, *p*=0.037). In a third sample (n=10) of patients with TRS treated with clozapine, IL-1β was correlated with passive/apathetic social withdrawal (*r*=0.657, *p*=0.039) and disturbance of volition (*r*=0.686, *p*=0.029) items of the Positive and Negative Symptom Scale and the global avolition-apathy scores of the Scale for the Assessment of Negative Symptoms (*r*=0.751, *p*=0.012). Finally, in a small subsample (n=5) of patients from this TRS cohort for whom we collected fMRI data, we found resting-state functional connectivity from a right nucleus accumbens seed to a cluster in medial prefrontal cortex. We found relationships between higher inflammation and decreased connectivity for TNF (*r*=−0.64) and CRP (*r*=−0.89). DISCUSSION/SIGNIFICANCE OF IMPACT: Taken together, these preliminary data show the predicted relationship between inflammatory markers and negative symptoms and demonstrate the reproducibility of TNF and other monocytic-derived cytokines as reliably elevated in schizophrenia and associated with negative symptoms across samples of patients with schizophrenia and individuals at high risk for psychosis. Cytokines may exert their effects via their impact on brain reward circuitry, and could represent novel treatment targets for motivational deficits and negative symptoms of schizophrenia.

